# Changing demographics and immunity to vaccine preventable diseases in people with HIV in Ireland

**DOI:** 10.1186/s12879-022-07487-z

**Published:** 2022-06-29

**Authors:** C. Kerr, M. Kelleher, S. Coughlan, B. Crowley, E. J. O’Reilly, C. Bergin

**Affiliations:** 1 Department of Infectious Diseases, St. James’s Hospital, Dublin, Ireland; 2 Department of Clinical Medicine, Trinity College Dublin, Dublin, Ireland; 3 Department of Microbiology, St. James’s Hospital, Dublin, Ireland; 4 National Virus Reference Laboratory, Belfield, Dublin, Ireland; 5 School of Public Health, University College Cork, Cork, Ireland

**Keywords:** Seroepidemiology, HIV, Measles, Mumps, Rubella, Varicella Zoster, Hepatitis A, Hepatitis B, Demographics

## Abstract

**Background:**

HIV infection is associated with an increased risk of morbidity and mortality from vaccine preventable infections. This research describes, in the context of changing patient demographics, the seroprevalence of vaccine preventable viral infections among attendees of the largest centre for HIV positive patients in Ireland.

**Methods:**

Baseline serum IgG results for measles, mumps, rubella, varicella zoster virus (VZV) & hepatitis A, as well as hepatitis B sAg, cAb and sAb results, were retrieved for 2534 clinic attendees attending in 2018. Results were available for between 990 and 2363 attendees (39–93%), depending on the test, and were compared with 2013 clinic data.

**Results:**

There was a 35% increase in attendees in 2018 when compared to 2013. The largest increase was in attendees of South American origin. In 2018, males accounted for 73% of the entire cohort and the HIV acquisition risk for 48% of attendees was MSM. 47% of attendees were originally from Ireland. Among those tested, 33% were susceptible to at least one component of the MMR vaccine. 5% were VZV non-immune (significantly associated with younger age and the acquisition risk status of injection drug use). 21% were hepatitis A non-immune (significantly associated with younger age and being of European or South American origin). 32% were hepatitis B cAb seropositive (significantly associated with older age, injection drug use status and being originally from Africa). 3% demonstrated hepatitis B sAg positivity. 64% had hepatitis B sAb ≥ 10mIU.

**Conclusion:**

In a cohort of attendees to an HIV clinic in a large urban setting, the susceptibility to several common vaccine preventable viral infections, in particular MMR and hepatitis A and B, was high. These results highlight the importance of proactive screening and immunisation to help protect this high risk patient group against vaccine preventable diseases.

## Background

Despite the development of effective therapies, people living with HIV (PLWH) still remain more at risk of infection and complications from vaccine preventable infections in comparison to the general population [1–4]. As a result, international guidelines for the care of those living with HIV universally have a lower threshold to recommend vaccination [5–10].

Several vaccine preventable viruses can cause concomitant infection and lead to severe morbidity and death among PLWH. Measles classically presents with fever, malaise, cough, coryza and conjunctivitis, and is an extremely contagious virus that remains a major cause of mortality worldwide. Immunosuppressed attendees such as PLWH are more at risk of developing severe measles infection and associated complications such as pneumonia and encephalitis. Mumps can present with fever, headache, myalgia and parotitis and can lead to complications such as meningitis, encephalitis, deafness, orchitis in males and oophoritis in females. Rubella usually results in a mild or even subclinical infection with symptoms that can consist of a rash and low-grade fever sometimes accompanied by arthritis or arthralgias. However, infection in pregnancy can cause spontaneous abortion, stillbirth or congenital rubella syndrome, leaving new born children with devastating neurological defects. Varicella zoster virus (VZV) typically causes a self-limiting illness. However, it can cause severe infection in adults and the immunocompromised, leading to disseminated varicella and complications such as encephalitis, pneumonitis and even death.

Hepatitis A virus is the commonest cause of viral hepatitis globally. It is spread through the faecal oral route by contaminated food or water. There have been recent outbreaks seen in the MSM (men who have sex with men) community [11]. The disease is usually self-limiting but can cause fulminant hepatic failure in a minority of cases. It is estimated that two billion people worldwide have been infected with the hepatitis B virus in their lifetime, with approximately 250 million being chronic carriers of the infection. Its prevalence is highest in Africa, Asia, South America, and Eastern Europe. Its modes of transmission include vertical as well as through blood transfusion, percutaneous inoculation, and sexual transmission. Fulminant liver failure is rare however cirrhosis can occur in chronic carriers, with a further risk of development of hepatocellular carcinoma irrespective of the development of cirrhosis. Those with HIV and hepatitis B coinfection are at an increased risk for this progression.

The combination MMR vaccine has been instrumental in reducing the number of cases of measles, mumps and rubella globally since its introduction in the 1960s. MMR vaccination is now part of routine childhood vaccination programmes worldwide, including in Ireland [9], however outbreaks of the viruses still exist in developing countries whose vaccination programmes are not well established [12–15] and in developed countries where childhood immunity responses may wane [16–18]. A varicella vaccine exists though it is not part of the routine childhood vaccination programme in Ireland [9]. Stand-alone vaccinations to protect against hepatitis A (inactivated virus vaccine) and hepatitis B (recombinant vaccine) infection, as well as a combined hepatitis A and B vaccine, are also available. In Ireland, the hepatitis A vaccine is currently indicated for international travellers to certain countries with high hepatitis A endemicity, those with hepatitis B, C and HIV infection and MSM. Hepatitis B vaccination entered the Irish childhood vaccination schedule in 2008. Prior to this, the vaccine was recommended for certain high risk groups such as healthcare workers, those with hepatitis C and HIV, travellers to countries with high levels of hepatitis B endemicity and those who live with someone with chronic hepatitis B. The national immunisation advisory committee (NIAC) in Ireland currently recommend that people living with HIV should generally receive all vaccines now recommended to the general population (such as MMR and hepatitis B vaccination) and some additional vaccines (including varicella and hepatitis A vaccination).

St James’s Hospital, Dublin, is home to the largest outpatient HIV clinic in Ireland, catering to over half of PLWH in the country. The clinic boasts a dedicated, in-house, vaccine unit for attendees. Previous research by Sadlier et al. examined baseline hepatitis A, hepatitis B and VZV serology in this cohort in 2013 [19]. Our research aims to examine the baseline seroprevalence of vaccine preventable viral infections among all PLWH, influenced by changing attendee demographics, and to compare these findings to those of Sadlier et al. 5 years previous.

## Methods

Ethical approval for this study was granted by the local St. James’s Hospital/Tallaght University Hospital research ethics committee (REC: 2020-02 List 7).

All 2018 attendees of the HIV outpatient clinic of St. James’s Hospital, Dublin, were included in this retrospective review. The baseline/earliest-available measles IgG, mumps IgG, rubella IgG, VZV IgG, hepatitis A IgG, hepatitis B sAg, cAb and sAb results from 2009 to 2018 inclusive were retrieved from laboratory databases and the electronic patient records of attendees. Baseline hepatitis B sAb titres of ≥ 10 mIU were considered to denote immunity, and titres less than this were considered non-immune. For the purposes of analysis, equivocal serologies were interpreted as demonstrating immunity/seropositivity/exposure.

Demographic data were retrieved from electronic attendee records. Mode of HIV acquisition was divided into the following categories: Heterosexual, MSM, people who inject drugs (PWID) and Other. Vertical transmission, contaminated blood products, needlestick injury and unknown risk were classed together as “other”.

Data were analysed using MS Excel version 16 and Stata version 14 for Windows. Categorical data are reported as count and relative frequency whereas continuous data is described as median and interquartile range. Between-group prevalence was compared using the Chi-square test and Wilcoxon signed-rank test. P values were considered significant if < 0.05. Univariate variables with p < 0.2 were entered into a multivariate logistic regression model for further analysis.

## Results

The records of the 2,534 individual attendees who attended the HIV outpatient clinic between January 1st and December 31st, 2018, were included in this study. Table 1 shows the baseline demographics of the attendees.

**Table 1:** Baseline attendee demographics of the entire cohort

2018 attendees	n	%	Median age (IQR)	Male	%	Female	%
**Total**	2534		42 years (35–50)				
Male	1854	73	42 years (35–51)				
Female	680	27	42 years (37–49)				
**HIV Acquisition Risk**							
Men who have sex with men (MSM)	1220	48	39 years (33–49)	1218	100	2	0
Heterosexual	907	36	44 years (38–51)	352	39	555	61
People who inject drugs (PWID)	359	14	44 years (39–52)	256	71	103	29
Other*	48	2	31 years (25–49)	28	58	20	42
**Region of Origin**							
Ireland	1193	47	46 years (38–54)	978	82	215	18
Africa	554	22	44 years (37–49)	192	35	362	65
Europe	380	15	39 years (35–46)	308	81	72	19
South America	280	11	33 years (30–37)	269	96	11	4
Asia and Australasia	87	3	43 years (33–49)	69	79	18	21
North & Central America	40	2	43 years (31–54)	38	95	2	5

*Vertical transmission, contaminated blood products, needlestick injury and unknown risk were classed together as “Other”

The cohort comprised of 1854 male (73%) and 680 (27%) female attendees. Attendees ranged in age from 18 to 84 years. The median age was 42 years with an interquartile range of 35–50 years. Of the 680 female attendees, 362 (53%) were originally from Africa.

Regarding mode of HIV acquisition, MSM accounted for the largest cohort of attendees at 1220 (48% overall and 66% of men), followed by heterosexual acquisition 907 (36%), injection drug use 359 (14%) and the remaining 2% of cases comprising of 23 cases of infection acquisition from vertical transmissions, 15 from contaminated blood products, 8 from needlestick injuries and 2 with unknown modes of acquisition. Two attendees whose HIV acquisition risk was classed as MSM identified as female.

The median age of attendees whose mode of HIV acquisition was defined as heterosexual or PWID (people who inject drugs) was 44 years while in those whose acquisition status was defined as MSM or Other it was 39 and 31 years, respectively. Median age differed by region of origin. The region with the oldest median age of attendees was Ireland at 46 years. The region with the youngest median age of attendees was South America at 33 years.

Table 2 compares the census of attendees attending the clinic in 2013 with the clinic’s 2018 attendee census. The clinic cohort grew by more than one third (35%) between 2013 and 2018 (Table 2). The MSM cohort increased by 495 (68%) during this time. By 2018, 48% of attendees identified as MSM compared to 39% in 2013. There was a substantial increase in attendees from South America (increased by 284%), North and Central America (increased by 122%) and Europe (increased by 62%), with South American attendees accounting for more than 10% of all attendees in 2018 (this figure was 4% in 2013). MSM was the identified acquisition risk for 84% of the South American cohort. 1,193 (47%) of attendees in 2018 were originally from Ireland, 554 (22%) from Africa, 373 (15%) from Europe, 280 (11%) from South America, 87 (3%) from Asia and Australasia (3%) and 40 (2%) from North & Central America.

**Table 2:** Change in demographics of clinic attendees in 2013 compared to 2018 attendees

	2013 attendees	%	2018 attendees	%	Overall change in attendees from 2013 to 2018	Change as a % of the 2013 demographic	Change as a % of the overall 2013 cohort
Total	1881		2534		653		+ 35
Median age (years)	40		42				
IQR (years)	33–47		35–50				
Male	1275	68	1854	73	579	+ 45	+ 31
Female	606	32	680	27	74	+ 12	+ 4
HIV acquisition risk							
MSM	725	39	1220	48	495	+ 68	+ 26
Heterosexual	754	40	907	36	153	+ 20	+ 8
PWID	367	20	359	14	− 8	− 2	0
Other	35	2	48	2	13	+ 37	+ 1
Region of Origin							
Ireland	1023	54	1193	47	170	+ 17	+ 9
Africa	469	25	554	22	85	+ 18	+ 5
Europe	234	12	380	15	146	+ 62	+ 8
South America	73	4	280	11	207	+ 284	+ 11
Asia and Australasia	64	3	87	3	23	+ 36	+ 1
North & Central America	18	1	40	2	22	+ 122	+ 1

Table 3 examines the demographics of 2018 attendee attendees by year of their first clinic attendance. Attendees were split into two groups; (1) attendees who first attended between 1987 (when the clinic was founded) and 2013 inclusive, and (2) attendees who first attended between 2014 and 2018 inclusive. Those who first attended from 2014 onwards were older at their first visit compared to those who first attended from 1987 to 2013 (mean age 35.7 vs. 32.9 years, p < 0.001). A higher proportion of attendees who first attended from 2014 onwards were male (84 vs. 66%, p < 0.001). There was a significant increase in attendees whose acquisition risk was identified as MSM (68 vs. 36%, p < 0.001), but a decrease in those whose acquisition risk was heterosexual (25% v 42%, p < 0.001) and PWID (5 vs. 20%, p < 0.001). No significant change was seen in those from the “other” category.

**Table 3:** Age, gender and acquisition risk factors of 2018 attendees, analysed by their date of first clinic attendance—sub-analysis

Variable						P value
**Age at first attendance**						
1987–2013	Mean age–32.9 yrs	SD (9.1 yrs)	Median age–31 yrs	IQR (26–38 yrs)		
2014–2018	Mean age–35.7 yrs	SD (9.7 yrs)	Median age–34 yrs	IQR (29–41 yrs)		< 0.001
**Gender**	**Male**	**%**	**Female**	**%**	Total	
1987–2013	1043	66	528	34	1571	
2014–2018	811	84	152	16	963	
Overall	1854	73	680	27	2534	< 0.001
**Acquisition risk**	**No**	**%**	**Yes**	**%**		
**MSM**						
1987–2013	1005	64	566	36	1571	
2014–2018	309	32	654	68	963	
Overall	1314	52	1220	48	2534	< 0.001
**Heterosexual**						
1987–2013	909	58	662	42	1571	
2014–2018	718	75	245	25	963	
Overall	1627	64	907	36	2534	< 0.001
**PWID**						
1987–2013	1260	80	311	20	1571	
2014–2018	915	95	48	5	963	
Overall	2175	86	359	14	2534	< 0.001
**Other**						
1987–2013	1539	98	32	2	1571	
2014–2018	947	98	16	2	963	
Overall	2486	98	48	2	2534	0.501

Table 4 explores the change in reported regions of origin of 2018 attendees over time by year of first clinic attendance. In comparison to attendees who first attended between 1987 and 2013 inclusive, attendees whose first attendance was from 2014 onwards were less likely to be originally from Ireland (32 vs. 56%, p < 0.001), and Africa (15 vs. 26%, p < 0.001), but more likely to be from South America (25 vs. 3%, p < 0.001), Europe (20 vs. 12%, p < 0.001), Asia and Australasia (5 vs. 3%, p = 0.007) and North America (3 vs. 1%, p < 0.001).

**Table 4:** Region of origin of 2018 attendees, analysed by their date of first clinic attendance—sub-analysis

Variable	No	%	Yes	%		P value
**Region of Origin**						
**Ireland**						
First attendance 1987–2013	689	44	882	56	1571	
First attendance 2014–2018	652	68	311	32	963	< 0.001
**Europe**						
First attendance 1987–2013	1383	88	188	12	1571	
First attendance 2014–2018	771	80	192	20	963	< 0.001
**Africa**						
First attendance 1987–2013	1164	74	407	26	1571	
First attendance 2014–2018	816	85	147	15	963	< 0.001
**South America**						
First attendance 1987–2013	1530	97	41	3	1571	
First attendance 2014–2018	724	75	239	25	963	< 0.001
**Asia and Australasia**						
First attendance 1987–2013	1529	97	42	3	1571	
First attendance 2014–2018	918	95	45	5	963	0.007
**North America**						
First attendance 1987–2013	1560	99	11	1	1571	
First attendance 2014–2018	934	97	29	3	963	< 0.001

Table 5 shows the available baseline measles (n = 990), mumps (n = 998), rubella (n = 997) and VZV (n = 1522) IgG serology results of our clinic attendees, along with univariate analysis of these figures. As can be seen from this table, a significant proportion of attendees were seronegative at baseline for measles (16%), mumps (17%) and rubella IgG (11%). 329 (33%) of attendees were seronegative for one or more of measles, mumps and rubella IgG. 5% of the cohort with available results were seronegative for VZV IgG.

**Table 5:** Breakdown of measles, mumps, rubella and VZV IgG results with associated univariate variable p values

	Measles IgG (n = 990)					Mumps IgG (n = 998)					Rubella IgG (n = 997)					VZV IgG (n = 1522)				
	Positive	%	Negative	%	P value	Positive	%	Negative	%	P value	Positive	%	Negative	%	P value	Positive	%	Negative	%	P value
Overall	827	84	163	16		827	83	171	17		884	89	113	11		1452	95	70	5	
Gender (M/F)	655/172	82/90	144/19	18/10	0.007	663/164	82/86	144/27	18/16	0.221	716/168	90/85	83/30	10/15	0.058	1127/325	96/94	49/21	4/6	0.137
MSM	502	80	128	20	< 0.001	511	81	123	19	0.012	557	88	73	12	0.741	841	96	38	4	0.548
Heterosexual	270	92	25	8	< 0.001	258	87	40	13	0.042	269	89	33	11	0.789	479	95	23	5%	0.982
PWID	44	92	4	8	0.119	47	96	2	4	0.013	46	96	2	4	0.108	103	93	8	7	0.173
Other	11	65	6	35	0.035	11	65	6	35	0.045	12	71	5	29	0.018	29	97	1	3	0.738
Ireland	293	93	21	7	< 0.001	273	85	47	15	0.159	292	92	24	8	0.011	567	97	18	3	0.025
Europe	139	75	47	25	< 0.001	153	82	34	18	0.673	166	88	22	12	0.86	267	95	15		0.523
Africa	170	91	16	9	0.001	168	90	19	10	0.005	166	88	22	12	0.86	288	95	15	5%	0.744
South America	156	68	73	32	< 0.001	174	76	55	24	0.002	201	88	28	12	0.627	239	94	15	6	0.276
Asia & Australasia	47	96	2	4	0.017	41	84	8	16	0.878	40	80	10	20	0.047	62	91	6	9	0.089
North America	22	85	4	15	0.88	18	69	8	31	0.062	19	73	7	27	0.011	29	97	1	3	0.738
Median age in years (IQR)	38 (32—46)		32 (29—36)		< 0.001	37 (32—45)		34 (29—40)		< 0.001	37 (32—45)		33 (29—39)		< 0.001	39 (33—47)		37 (31—41)		0.008

On univariate analysis, male gender (p = 0.007), MSM (p < 0.001), belonging to the “other” acquisition risk category group (p = 0.035), being originally from Europe (p < 0.001) and South America (p < 0.001) were associated with measles IgG seronegativity. Female gender (p = 0.007), heterosexual acquisition risk category (p < 0.001), being originally from Ireland (p < 0.001), Africa (p < 0.001), Asia & Australasia (p = 0 0.017) and older age (p < 0.001) were associated with measles IgG seropositivity. MSM acquisition risk (p = 0.012), being in the “other” acquisition risk category (p = 0.045) and being originally from South America (p = 0.002) were associated with mumps IgG seronegativity on univariate analysis. Belonging to the heterosexual acquisition risk category (p = 0.042), PWID status (p = 0.013), being originally from Africa (p = 0.005) and older age (p < 0.001) were associated with mumps IgG positivity. Belonging to the “other” acquisition risk category (p = 0.018), being originally from North America (p = 0.011) and Asia & Australasia (p = 0.047) were associated with rubella IgG seronegativity on univariate analysis. Being originally from Ireland (p = 0.011) and older age (p < 0.001) were associated with rubella IgG seropositivity. Being originally from Ireland (p = 0.025) and older age (p = 0.008) were associated with VZV IgG seropositivity on univariate analysis.

Table 6 examines measles, mumps, rubella and VZV IgG seropositivity of 2018 attendees, stratified into two groups; those whose first attendance was between 1987 and 2013 inclusive, and those whose first attendance was between 2014 and 2018 inclusive. Those who first attended from 2014 onwards were significantly less likely to exhibit measles IgG positivity (81 vs. 94%, p < 0.001), but more likely to exhibit VZV IgG positivity (96% v 94%, p = 0.045). No significant difference was seen in mumps IgG or rubella IgG seropositivity.

**Table 6:** Measles, mumps, rubella and VZV IgG serology results of 2018 attendees, analysed by their date of first clinic attendance–sub-analysis

Variables	Positive	%	Negative	%	Total	P value
**Measles IgG**						
First attendance 1987–2013	195	94	13	6	208	
First attendance 2014–2018	632	81	150	19	782	< 0.001
**Mumps IgG**						
First attendance 1987–2013	188	87	28	13	216	
First attendance 2014–2018	639	82	143	18	782	0.066
**Rubella IgG**						
First attendance 1987–2013	196	91	19		215	
First attendance 2014–2018	688	88	94	12	782	0.192
**VZV IgG**						
First attendance 1987–2013	693	94	42		735	
First attendance 2014–2018	759	96	28	4	787	0.045

Univariate variables with p < 0.2 were entered into a multivariate logistic regression model for further analysis. The acquisition risk category “other” was omitted from Measles IgG and Mumps IgG multivariate analysis due to collinearity. On multivariate analysis (Table 9), older attendees were less likely to exhibit seronegativity for measles IgG (OR 0.91, 95% CI 0.88–0.94, p < 0.001), mumps IgG (OR 0.97, 95% CI 0.95–0.99, p = 0.001), rubella IgG (OR 0.96, 95% CI 0.94–0.98, p = 0.001) and VZV IgG (OR 0.96, 95% CI 0.94–0.99, p = 0.007). Age was the only statistically significant variable seen on multivariate analysis for measles IgG among the measured variables. MSM (OR 0.29, 95% CI 0.09–0.91, p = 0.034) and PWID attendees (OR 0.07, 95% CI 0.01–0.45, p = 0.005) were significantly less likely to exhibit mumps IgG seronegativity on multivariate analysis, along with attendees of African origin (OR 0.42, 95% CI 0.21–0.82, p = 0.012). Gender was significantly associated with rubella IgG results, with female attendees more likely to be seronegative compared to their male counterparts (OR 1.63, 95% CI 1.02–2.61, p = 0.042). Those of North American (OR 3.37, 95% CI 1.34–8.47, p = 0.010) and Asian & Australasian origin (OR 2.23, 95% CI 1.05–4.72, p = 0.036) origin were more likely to exhibit Rubella IgG seronegativity. In relation to VZV IgG status, those whose HIV acquisition risk was deemed to be PWID (OR 3.11, 95% CI 1.32–7.31, p = 0.009) were more likely to exhibit VZV IgG seronegativity.

Table 7 shows results and univariate analysis for available hepatitis A IgG, hepatitis B sAg, cAb and sAb results.

**Table 7:** Breakdown of hepatitis A IgG, hepatitis B sAg, hepatitis B cAb and hepatitis C Ab results with associated univariate variable p values

	Hepatitis A IgG (n = 2294)					Hepatitis B sAg (n = 2322)					Hepatitis B cAb (n = 1879)					Hepatitis B sAb ≥ 10mIU (n = 2363)				
	Positive	%	Negative	%	P value	Positive	%	Negative	%	P value	Positive	%	Negative	%	P value	Positive	%	Negative	%	P value
Overall	1806	79	488	21		63	3	2259	97		610	32	1269	68		1501	64	862	36	
Gender (M/F)	1287/519	75/88	420/68	25/12	< 0.001	46/17	3/3	1673/586	97/97	0.852	431/179	30/40	997/272	70/60	< 0.001	1146/355	66/58	602/260	34/42	0.001
MSM	813	70	341	30	< 0.001	16	1	1124	99	< 0.001	244	25	733	75	< 0.001	825	71	339	29	< 0.001
Heterosexual	700	87	105	13	< 0.001	38	5	775	9	< 0.001	235	38	388	62	0.001	464	56	362	44	< 0.001
PWID	263	89	31	11	< 0.001	8	2	316	98	0.771	122	50	120	50	< 0.001	186	56	144	44	0.004
Other	30	73	11	27	0.431	1	2	44	9	0.838	9	24	28	76	0.294	26	60	17	40	0.674
Ireland	848	81	198	19	0.015	12	1	1060	99	< 0.001	233	29	578	71	0.004	712	63	424	37	0.412
Europe	235	65	128	35	< 0.001	14	4	343	96	0.127	111	34	213	66	0.46	230	65	123	35	0.489
Africa	471	97	17	3	< 0.001	29	6	469	94	< 0.001	182	48	198	52	< 0.001	295	60	200	40	0.041
South America	161	58	117	42	< 0.001	2	1	272	99	0.031	55	21	209	79	< 0.001	184	70	78	30	0.017
Asia & Australasia	65	79	17	21	0.812	5	6	80	94	0.067	23	33	47	67	0.92	58	71	24	29	0.167
North America	26	70	11	30	0.239	1	3	35	97	0.981	6	18	27	82	0.8	22	63	13	37	0.934
Median age in years (IQR)	43 (36—51)		36 (30—43)		< 0.001	45 (38—49)		41 (35—50)		0.148	45 (38—53)		38 (32—46)		< 0.001	42 (35—50)		41 (35—50)		0.772

21% of 2018 attendees with available baseline Hepatitis A IgG results (n = 2294) were seronegative (Table 7). Male gender (p < 0.001), MSM (p < 0.001), being originally from Europe (p < 0.001) and South America (p < 0.001) were associated with hepatitis A IgG seronegativity on univariate analysis. Female gender (p < 0.001), PWID (p < 0.001), heterosexuals (p < 0.001), being originally from Ireland (p = 0.015), from Africa (p < 0.001) and older age (p < 0.001) were associated with hepatitis A IgG seropositivity. 3% of the 2322 attendees with available baseline Hepatitis B sAg results were positive for Hepatitis B sAg. MSM (p < 0.001), being originally from Ireland (p < 0.001) and South America (p = 0.031) were associated with hepatitis B sAg seronegativity on univariate analysis. Heterosexual status (p < 0.001) and being originally from Africa were significantly associated with hepatitis B sAg seropositivity. Of attendees with available baseline hepatitis B cAb results (n = 1879), 32% exhibited seropositivity. Male gender (p < 0.001), MSM (p < 0.001), being originally from Ireland (p = 0.004) and South America (p < 0.001) were associated with hepatitis B cAb seronegativity on univariate analysis. Female gender (p < 0.001), heterosexual status (p = 0.001), PWID (p < 0.001), being from Africa (p < 0.001) and older age (p < 0.001) were associated with hepatitis B cAb seropositivity. 64% of clinic attendees were classed as having hepatitis B sAb results ≥ 10mIU. Female gender (p = 0.001), heterosexual status (p < 0.001), PWID (p = 0.004) and being from Africa was associated with hepatitis B sAb levels < 10mIU. Male gender (p = 0.001), MSM status (p < 0.001) and being from South America (p = 0.017) was associated with hepatitis B sAb levels ≥ 10mIU.

Table 8 examines the 2018 attendees when they are split into two groups: those who first attended the clinic from its inception in 1987 up to and including 2013, and those who first attended the clinic from 2014 onwards. The analyses in this table demonstrates how 2018 attendees who first attended the clinic from 2014 onwards were less likely to exhibit hepatitis A IgG positivity (66% v 88%, p < 0.001), less likely to demonstrate hepatitis B sAg positivity (2% v 4%, p = 0.026) and less likely to demonstrate hepatitis B cAb positivity (25% v 39%, p < 0.001) in comparison to those who first attended the clinic between 1987 and 2013. No statistically significant difference was exhibited among the two groups in terms of hepatitis B sAb response, with a similar proportion of both groups demonstrating hepatitis B sAb ≥ 10mIU (65% v 62%, p = 0.167).

**Table 8:** Hepatitis A IgG, hepatitis B sAg, hepatitis B cAb and hepatitis b sAb results from 2018 attendees, analysed by their date of first clinic attendance—sub-analysis

Variables	Positive	%	Negative	%	Total	P value
**Hepatitis A IgG**						
First attendance 1987–2013	1188	88	168	12	1356	
First attendance 2014–2018	618	66	320	34	938	< 0.001
**Hepatitis B sAg**						
First attendance 1987–2013	46	4	1135	96	1181	
First attendance 2014–2018	17	2	924	98	941	0.026
**Hepatitis B cAb**						
First attendance 1987–2013	381	39	584	61	965	
First attendance 2014–2018	229	25	685	75	914	< 0.001
**Hepatitis B sAb**	≥ 10mIU	%	< 10mIU	%	Total	
First attendance 1987–2013	957	65	525	35	1482	
First attendance 2014–2018	544	62	337	38	881	0.167

**Table 9:** Multivariate analysis of serological results incorporating univariate variables with p < 0.2

	Odds ratio	95% Confidence interval		P value
**Hepatitis A IgG negativity**				
Female gender	0.75	0.50	1.11	0.146
Increasing age (by year)	0.94	0.93	0.96	< 0.001
MSM	0.51	0.22	1.17	0.111
Heterosexual	0.61	0.26	1.42	0.254
PWID	0.23	0.09	0.57	0.001
Ireland	1.10	0.69	1.77	0.682
Europe	1.84	1.13	3.01	0.015
Africa	0.12	0.06	0.24	< 0.001
South America	1.67	1.00	2.76	0.048
**Hepatitis B sAg positivity**				
Increasing age (by year)	1.02	0.99	1.05	0.190
MSM	0.43	0.18	1.06	0.066
Heterosexual	0.83	0.35	1.95	0.664
Ireland	0.28	0.03	2.26	0.231
Europe	1.26	0.16	10.02	0.825
Africa	1.31	0.16	10.57	0.801
South America	0.33	0.03	3.80	0.373
Asia and Australasia	1.91	0.21	17.20	0.562
**Hepatitis B cAb positivity**				
Female gender	1.09	0.81	1.48	0.572
Increasing age (by year)	1.06	1.05	1.08	< 0.001
MSM	1.43	0.61	3.35	0.405
Heterosexual	1.01	0.44	2.31	0.985
PWID	4.60	1.92	11.01	0.001
Ireland	0.44	0.33	0.59	< 0.001
Africa	2.28	1.56	3.31	< 0.001
South America	0.84	0.57	1.23	0.361
**Hepatitis B sAb < 10mIU**				
Female gender	0.91	0.72	1.16	0.454
MSM	0.55	0.29	1.04	0.068
Heterosexual	1.23	0.65	2.30	0.525
PWID	1.05	0.54	2.04	0.878
Africa	0.81	0.62	1.05	0.109
South America	0.97	0.72	1.31	0.854
**Measles IgG negativity**				
Female gender	0.84	0.37	1.90	0.675
Increasing age (by year)	0.91	0.88	0.94	< 0.001
MSM	0.53	0.15	1.87	0.323
Heterosexual	0.42	0.13	1.42	0.165
PWID	0.65	0.12	3.42	0.612
Other	1.00			
Ireland	0.45	0.14	1.51	0.196
Europe	2.14	0.67	6.83	0.200
Africa	0.64	0.17	2.41	0.511
South America	1.85	0.59	5.78	0.291
Asia and Australasia	0.23	0.04	1.39	0.108
**Mumps IgG negativity**				
Increasing age (by year)	0.97	0.95	0.99	0.001
MSM	0.29	0.09	0.91	0.034
Heterosexual	0.35	0.11	1.06	0.064
PWID	0.07	0.01	0.45	0.005
Other	1.00			
Ireland	0.93	0.59	1.49	0.770
Africa	0.42	0.21	0.82	0.012
South America	1.26	0.79	2.03	0.332
North America	2.09	0.84	5.20	0.111
**Rubella IgG negativity**				
Female gender	1.63	1.02	2.61	0.042
Increasing age (by year)	0.96	0.94	0.98	0.001
PWID	0.53	0.12	2.28	0.391
Other	2.10	0.69	6.42	0.193
Ireland	0.80	0.48	1.32	0.376
Asia and Australasia	2.23	1.05	4.72	0.036
North America	3.37	1.34	8.47	0.010
**VZV IgG negativity**				
Female gender	1.47	0.86	2.51	0.163
Increasing age (by year)	0.96	0.94	0.99	0.007
PWID	3.11	1.32	7.31	0.009
Ireland	0.56	0.30	1.04	0.067
Asia and Australasia	1.98	0.81	4.87	0.135

On multivariate analysis (Table 9), older clinic attendees (OR 0.94, 95% CI 0.93–0.96, p < 0.001), attendees whose HIV acquisition risk was classed as PWID (OR 0.23, 95% CI 0.09–0.57, p = 0.001) and those originally of African origin (OR 0.12, 95% CI 0.06–0.24, p < 0.001) were significantly less likely to exhibit hepatitis A IgG seronegativity. Being originally of European (OR 1.84, 95% CI 1.13–3.01, p = 0.015) and South American (OR 1.67, 95% CI 1.00–2.76, p = 0.048) origin was significantly associated with hepatitis A IgG seronegativity.

Multivariate analysis revealed statistically significant associations between seropositivity and increasing age (OR 1.06, 95% CI 1.05–1.08, P < 0.001), PWID status (OR 4.6, 95% CI 1.92–11.01, p = 0.001) and being originally of African origin (OR 4.6, 95% CI 1.92–11.01, p < 0.001). Those of Irish origin were statistically less likely to exhibit Hepatitis B cAb seropositivity (OR 0.44, 95% CI 0.33–0.59, P < 0.001).

No variables were significantly associated with hepatitis B cAb seropositivity or hepatitis B immunity on multivariate analysis.

## Discussion

This research highlights several notable features of the demographics and selected seroepidemiology of PLWH attending Ireland’s largest HIV clinic.

Firstly, these results show the male predominance of attendees at the clinic. This research also highlights the geographical diversity of the current HIV clinic attendees. Over 50% of the attendees are originally from regions outside of Ireland. The identified mode of HIV acquisition for almost half of the HIV positive cohort is MSM, followed by heterosexual acquisition in just over a third and injection drug use in less than a fifth of cases.

Attendees of Irish (median age 46) and African origin (median age 44) tended to be older in comparison to attendees originally from Europe (median age 39) and South America (median age 33). Those whose acquisition risks are heterosexual (median age 44) and PWID (median age 44) tended to be older than those whose acquisition risk is MSM (median age 39) or other (median age 31).

In 2013, the number of attendees attending the clinic for their HIV care was 1881. By 2018, this figure grew by more than a third to 2534. In 2013, the heterosexual cohort made up the largest cohort of attendees by likely mode of HIV acquisition at 40%. In 2018, the MSM cohort was the largest, having grown from 39 to 48%. Attendees from Ireland comprised more than half of the cohort (54%) in 2013. By 2018, attendees of Irish origin made up 47% of attendees, with the numbers of attendees from South America almost trebling in this time period to make up more than 10% of the overall cohort. These findings are reflected in national data which showed that those whose acquisition risk was MSM and those of South American origin made up 56% and 43% respectively of new diagnoses in Ireland in 2018 [20].

This research builds on previous work by Sadlier et al. in 2013. Sadlier et al. explored VZV IgG, hepatitis A IgG and combined hepatitis B sAg and cAb positivity of attendees of the clinic in 2013 (19). Our research shows similar levels of VZV IgG positivity (95% in our study versus 94% in the 2013 study by Sadlier et al.) and hepatitis B cAb seropositivity (32% in our study versus combined cAb/sAg positivity of 32% in 2013), with higher levels of hepatitis A IgG seropositivity seen in 2018 attendees compared to 2013 (79% in our study versus 75% in 2013).

Sub-analysis of serological results demonstrates how recent 2018 attendees (those who first attended from 2014 onwards) were less likely to demonstrate measles IgG, hepatitis A IgG, hepatitis B sAg and hepatitis B cAb positivity compared to earlier attendees who first registered to the clinic between 1987 and 2013. More recent attendees were, however, more likely to exhibit VZV IgG positivity. Given that VZV vaccination is not part of the routine childhood vaccination schedule in Ireland, this change is either due to vaccination of attendees originally from outside of Ireland or from increased levels of prior exposure to VZV among the new attendees. No statistically significant difference was seen between these two groups with regard to mumps IgG, rubella IgG or hepatitis B sAb results.

Sub-analysis of our 2018 attendees further demonstrates the shifting demographics of clinic attendees. Attendees who first registered to the clinic from 2014 onwards were older at first presentation, more likely to have an acquisition risk identified as MSM, and more likely to be from Europe, South America, North America and Asia and Australasia, when compared to attendees who first registered to the clinic between 1987 (when the clinic was founded) and 2013. Attendees from 2014 onwards were less likely to be originally from Ireland or Africa and less likely to have an HIV acquisition risk identified as heterosexual or PWID. No change was seen in the proportion of attendees whose acquisition risk category was identified as “other”.

This research highlights the levels of susceptibility to common vaccine preventable viral infections among this population. Multivariate analyses revealed, unsurprisingly, that older age was associated with a decreased likelihood of seronegativity for measles, mumps and rubella IgG. MSM and PWID status was significantly associated with a decreased likelihood of mumps seronegativity, as was being of African origin. Those of North American and Asian & Australasian origin were significantly more likely to exhibit rubella IgG seronegativity. Interestingly, female attendees were also significantly more likely to exhibit rubella IgG seronegativity than their male counterparts. Given the risk of congenital rubella syndrome, this finding highlights the importance of identifying susceptible attendees and vaccinating them as soon as identified. 990 attendees had combined measles, mumps and rubella IgG results available for analysis. 329 (33%) were susceptible to at least one infection protected against by the MMR vaccine. This data is similar to other European studies of PLWH (21, 22).

Susceptibility to varicella virus among this population was lower, when compared to MMR susceptibility, at 5%. However, the level of VZV susceptibility in the attendee cohort is higher than that of the general population in Ireland (1–2%) (23). Older age was significantly associated with a decreased likelihood of VZV IgG seronegativity, whereas PWID were significantly more likely to demonstrate seronegativity. VZV infection leads to more frequent complications in adults and is more likely to be fatal in immunocompromised attendees (24, 25). Early identification and appropriate vaccination of susceptible individuals is crucial in avoiding significant morbidity and mortality among PLWH, especially in a cohort of attendees who may be more socioeconomically disadvantaged and may have difficulties in healthcare engagement.

Susceptibility to hepatitis A infection among this PLWH cohort is lower than what has been previously described (33%) in the general Irish population (26). Hepatitis A IgG seronegativity was significantly less likely to be seen in older attendees and those whose HIV acquisition status was PWID, possibly due to prior vaccination though exposure to infection is also a possibility. Attendees of African origin were significantly less likely to be seronegative for hepatitis A IgG, possibly due to the fact that historically, at least, the infection has been endemic to several countries on the African continent (27–29). Younger attendees and those of European origin and South American origin were significantly more likely to demonstrate hepatitis A IgG seronegativity, an important point to note in light of recent hepatitis A outbreaks in the MSM community worldwide, and due to the changing demographics of our clinic attendees with greater numbers of younger males of South American origin identifying as MSM attending the clinic in recent years. Current national vaccination guidelines recommend administration of the vaccine to those at risk including MSM, PWID and those travelling to endemic areas, as well as to all PLWH.

3% of the cohort tested positive for hepatitis B surface antigen, indicating current hepatitis B infection. None of the variables examined on multivariate analysis demonstrated any significant trends. 32% of attendees exhibited hepatitis B cAb seropositivity, indicating infection by the hepatitis B virus at some time. There were statistically significant associations between seropositivity and age, PWID status and being of African origin. This geographical association reflects the higher levels of hepatitis B endemicity in these regions (30), and the association with PWID status likely reflects the high rate of hepatitis B transmission associated with unsafe injection practices. Those of Irish origin were statistically more likely to demonstrate cAb seronegativity, which is not surprising given the low prevalence of hepatitis B in Ireland. Over 60% of attendees demonstrated hepatitis B sAb titres ≥ 10mIU at baseline, with no statistically significant variables identified on multivariate analysis.

Our study is limited by the lack of easily accessible electronic serological results for attendees pre-2009. While some serological tests, in particular hepatitis B sAg and sAb, tend to be tested regularly among high-risk groups such as PLWH, other tests such as measles, mumps and rubella serology tend to be checked at the attendee’s first clinic visit, with vaccination being organised as required at subsequent visits. Consequently, attendees who have been attending the clinic pre-2009 only have post-2009 serological results available for this analysis. Attendees’ vaccination data was not accessed as part of this study, and this is another limitation of the study. In addition, this study does not take into account the issue of waning immunity to these vaccine preventable viral infections. These data also do not capture serological results from outside our clinic, which can be an issue for attendees who have transferred their care from abroad. These issues continue to lend voice to the call for universal vaccine passports, a step which would allow for greater recording and knowledge of individual immunisation status. Such vaccine passports could be helpful in allowing healthcare workers to confirm vaccine receipt among patients for infections that currently don’t routinely undergo routine serology testing, such as HPV and pneumococcal infection.

## Conclusion

This research describes the changing demographics of Ireland’s largest HIV positive attendee cohort. It highlights the significant susceptibility of the cohort to several common vaccine preventable viruses, in particular MMR and hepatitis A and B. It has also highlighted how the susceptibility to these viruses is associated with the changing demographics of this cohort. The demographic findings in this research reflect changes in national HIV data. The associated vaccine susceptibility findings will inform not just local but also national vaccination practice. Further research is needed to examine the subsequent levels of vaccine receipt in this cohort. The widespread adoption of a vaccine passport would aid in ensuring that healthcare providers are more informed of their attendees’ vaccine history, especially in instances of patients transferring their care from one region to another.This research will help educate both healthcare staff and clinic attendees on the rates of susceptibility to these vaccine preventable viral infections, and in doing so, will promote vaccination discussion and referral.

## Data Availability

The datasets used and/or analysed during the current study are available from the corresponding author on reasonable request.
